# Brain Structural Signatures of Negative Symptoms in Depression and Schizophrenia

**DOI:** 10.3389/fpsyt.2014.00116

**Published:** 2014-08-27

**Authors:** Jie-Yu Chuang, Graham K. Murray, Antonio Metastasio, Nuria Segarra, Roger Tait, Jenny Spencer, Hisham Ziauddeen, Robert B. Dudas, Paul C. Fletcher, John Suckling

**Affiliations:** ^1^Department of Psychiatry, University of Cambridge, Cambridge, UK; ^2^Behavioural and Clinical Neuroscience Institute, University of Cambridge, Cambridge, UK; ^3^Cambridgeshire and Peterborough NHS Foundation Trust, Cambridge, UK; ^4^Norfolk and Suffolk NHS Foundation Trust, Norfolk, UK; ^5^Wellcome Trust MRC, Institute of Metabolic Science, University of Cambridge, Cambridge, UK

**Keywords:** negative symptoms, depression, schizophrenia, cerebellum, white matter

## Abstract

Negative symptoms occur in several major mental health disorders with undetermined mechanisms and unsatisfactory treatments; identification of their neural correlates might unveil the underlying pathophysiological basis and pinpoint the therapeutic targets. In this study, participants with major depressive disorder (*n* = 24), schizophrenia (*n* = 22), and healthy controls (*n* = 20) were assessed with 10 frequently used negative symptom scales followed by principal component analysis (PCA) of the scores. A linear model with the prominent components identified by PCA was then regressed on gray and white-matter volumes estimated from T1-weighted magnetic resonance imaging. In depressed patients, negative symptoms such as blunted affect, alogia, withdrawal, and cognitive impairment, assessed mostly via clinician-rated scales were inversely associated with gray matter volume in the bilateral cerebellum. In patients with schizophrenia, anhedonia, and avolition evaluated via self-rated scales inversely related to white-matter volume in the left anterior limb of internal capsule/anterior thalamic radiation and positively in the left superior longitudinal fasiculus. The pathophysiological mechanisms underlying negative symptoms might differ between depression and schizophrenia. These results also point to future negative symptom scale development primarily focused on detecting and monitoring the corresponding changes to brain structure or function.

## Introduction

Negative symptoms are prevalent, presenting in more than 50% of psychotic patients (1). Based on the analysis of the National Institute of Mental Health, Clinical Antipsychotic Trials of Intervention Effectiveness (CATIE) schizophrenia trial, negative symptoms have greater impact on functioning than positive or any other symptoms (2). Moreover, they have undetermined etiology and are resistant to treatment (3) with existing psychotropic drugs. Despite initially being thought to be restricted to schizophrenia, negative symptoms are also present in other major psychiatric disorders, notably major depressive disorder (4) with possibly different clinical presentations. For instance, anhedonia, one of the core domains within negative symptoms (5) appears to covary with clinical state in depressed patients but instead reflects an enduring trait in schizophrenia (6). Clinical differences such as these may imply distinct pathophysiology of negative symptoms in depression and schizophrenia, though as yet this remains uncertain.

Understanding more about the neuroanatomical correlates of negative symptoms in depression and schizophrenia may help elucidate whether negative symptoms in the two conditions represent the same phenomena or not. Although a number of studies have been conducted examining neural correlates of negative symptoms, the results have not been consistent and a variety of regions have been implicated, which prevented us from formulating an *a priori* hypothesis. In young women at high risk of depression, fractional anisotropy (FA) of bilateral cingulum bundles and left subgenual cingulum are negatively correlated with anhedonia (7). In depressed adolescents, FA was also found to be negatively correlated with anhedonia in anterior limb of internal capsule, posterior cingulum, and inferior-fronto-occipital fasciculus (8). In schizophrenia, negative symptoms inversely correlated with gray matter volume in cerebellum (9), left precuneus (10), right posterior cingulate (10); white-matter volume in regions near bilateral anterior cingulate and right internal capsule (11); FA in internal capsule (12), anterior thalamic radiation (12), parietal portion of the superior longitudinal fasciculus (12), fronto-occipital fasciculus (12), corpus callosum (12–14), medial frontal gyrus (15), and inferior frontal white matter (16). Positive correlation with negative symptoms in schizophrenia was also found in gray matter volume of right thalamus (17) and FA of an area near right insula (18), left cingulum and left superior longitudinal fasciculus II (19). However, some researchers have failed to identify any significant correlation between brain structure and negative symptoms (20, 21). Part of the reason for the heterogeneity of results may result from the heterogeneity in ways in which negative symptoms were measured. Many psychological scales have been devised for the evaluation of negative symptoms. In spite of the appreciable quantity of scales, individually, none covers all aspects of negative symptoms and none has been demonstrated to be superior in terms of severity evaluation or prognostic prediction.

In order to avoid selection bias, we decided to assess negative symptoms by using a large number of negative symptom scales, then employing a data reduction approach with PCA to identify the major dimensions of negative symptoms and specify their magnetic resonance imaging (MRI) anatomical correlates; namely gray and white-matter volumes estimated with voxel-based morphometry (VBM) (22). To our knowledge, this is the first study exploring negative symptoms in schizophrenia and major depressive disorder using integrative information from a large number of negative symptom scales. We hypothesize that distinct patterns of neural correlates will be associated with different domains of negative symptoms in depression and schizophrenia, respectively.

## Materials and Methods

### Participants and recruitment

Twenty-two participants with DSM-IV schizophrenia, 24 patients with DSM-IV major depressive disorder, and 20 control participants, were recruited to take part in the study (Table 1). Inclusion criteria were: adequate English proficiency and age between 18 and 65 years. Exclusion criteria were: brain structural abnormality, history of major medical or neurological disorder, substance dependence, and any contraindications to MRI scanning contraindications. First-degree family history of schizophrenia or bipolar disorder was an additional exclusion criterion for depressed and control subjects. The study was approved by the Cambridgeshire 3 National Health Service research ethics committee with written informed consent obtained from every participant.

**Table 1:** **Demographics and participants’ characteristics**.

	Control (±SD)	Depression (±SD)	Depression vs. control (*t* value/d.f.) *p* value	Schizophrenia (±SD)	Schizophrenia vs. control (*t* value/d.f.) *p* value
Number of subjects	20	24	–	22	–
Gender (women/men)	4/16	7/17	−0.69/42 0.50	3/19	0.54/40 0.59
Age (years)	34.30 ± 10.37 (range: 19–49)	33.08 ± 9.15 (range: 19–54)	0.41/42 0.68	32.73 ± 7.62 (range: 21–46)	0.56/40 0.576
Estimated IQ	115.35 ± 19.79 (range: 87–158)	107.08 ± 16.60 (range: 87–139)	1.51/42 0.14	99.38 ± 19.16 (range: 83–151)	2.63/39 0.01
Education (years)	14.79 ± 1.96 (range: 11–19)	13.43 ± 2.21 (range: 11–17)	2.08/40 0.04	13.62 ± 2.11 (range: 11–17)	1.81/38 0.08
Maternal education (years)	13.60 ± 2.77 (range: 9–19)	13.00 ± 1.65 (range: 11–16)	0.70/23.33 0.49	12.80 ± 2.83 (range: 9–19)	0.78/28 0.44
Handness	16 right 3 left 1 missing data	22 right 2 left	–	16 right 5 left 1 missing data	–
Ethnicity	16 white-British 1 Asian-Indian 1 other ethnicity 2 missing data	20 white-British 3 white-others 1 Asian-Indian	–	17 white-British 1 white-Irish 1 white-others 1 Asian-Indian 2 missing data	–
Current employment	13 working (paid) 3 unemployed 1 student 3 missing data	13 working (paid) 4 unemployed 2 student 2 others 3 missing data	–	4 working (paid) 3 voluntary 10 unemployed 1 other 4 missing data	–
Alcohol	1 none 5 <weekly 12 1–3 times/week 1 almost daily 1 missing data	5 none 1 1–2 times 8 <weekly 6 1–3 times/week 4 almost daily	–	2 none 1 1–2 times 3 <weekly 9 1–3 times/week 4 almost daily 3 missing data	–
Smoking	9 none 2 1–2 times 1 <weekly 7 almost daily 1 missing data	7 none 1 1–2 times 3 <weekly 13 almost daily	–	3 none 18 almost daily 1 missing data	–
Cannabis	7 none 3 1–3 times 7 <weekly 1 1–3 times/week 1 almost daily 1 missing data	11 none 4 1–2 times 5 <weekly 2 1–3 times/week 2 almost daily	–	4 none 2 1–3 times 4 <weekly 3 1–3 times/week 7 almost daily 2 missing data	–
HAMD-17 score (50)	0.65 ± 0.99 (range: 0–3)	16.33 ± 5.37 (range: 8–27)	−14.03/42 <0.000001	9.45 ± 5.54 (range: 2–20)	−7.33/40 <0.000001
PANSS total score	31.05 ± 2.37 (range: 30–39)	55.08 ± 14.60 (range: 35–98)	−7.94/42 <0.000001	58.14 ± 17.81 (range: 34–101)	−7.06/40 <0.000001
PANSS negative score	7.15 ± 0.37 (range: 7–8)	13.50 ± 6.19 (range: 7–33)	−5.02/42 0.000044	16.05 ± 5.41 (range: 9–31)	−7.69/40 <0.000001
SHAPS score	23.30 ± 3.66 (range: 15–29)	33.42 ± 6.81 (range: 15–41)	−6.27/42 <0.000001	28.86 ± 6.25 (range: 14–38)	−3.47/40 0.001
Chapman physical score	12.55 ± 9.07 (range: 2–34)	29.54 ± 14.11 (range: 4–51)	−4.83/42 0.000021	23.00 ± 9.45 (range: 4–35)	−3.65/40 0.001
Chapman social score	12.20 ± 7.02 (range: 3–32)	22.79 ± 10.94 (range: 3–39)	−3.88/42 0.000382	15.55 ± 7.55 (range: 6–32)	−1.48/40 0.146
MEI social score	28.90 ± 9.77 (range: 6–45)	16.17 ± 9.31 (range: 3–41)	4.42/42 0.000069	18.95 ± 6.08 (range: 1–26)	4.00/40 0.000264
MEI physical score	25.10 ± 7.50 (range: 11–39)	11.25 ± 6.11 (range: 3–25)	6.75/42 <0.000001	15.73 ± 6.24 (range: 0–25)	4.42/40 0.000075
TEPS anticipatory score	42.80 ± 8.43 (range: 20–55)	28.71 ± 8.44 (range: 11–53)	5.52/42 0.000002	35.36 ± 9.23 (range: 17–55)	2.72/40 0.01
TEPS consummatory score	37.15 ± 7.09 (range: 19–48)	26.58 ± 9.55 (range: 10–45)	4.09/42 0.000189	29.73 ± 7.09 (range: 21–48)	3.39/40 0.002
BDI anhedonia score	1.10 ± 1.37 (range: 0–4)	5.96 ± 2.26 (range: 2–11)	−8.41/42 <0.000001	4.91 ± 2.56 (range: 0–11)	−5.92/40 0.000001
BPRS negative symptom subscore	3	6.08 ± 3.27 (range: 3–17)	−4.62/42 0.00012	7.14 ± 2.64 (range: 3–14)	−7.34/40 <0.000001
Cognition/expression component	−0.87 ± 0.12 (range: −1.17 to −0.74)	0.17 ± 1.06 (range: −1.05–3.54)	−4.77/42 0.000077	0.61 ± 0.84 (range: −0.71–2.90)	−8.14/40 <0.000001
Pleasure/motivation component	−0.95 ± 0.64 (range: −2.06–0.25)	0.73 ± 0.84 (range: −1.44–1.93)	−7.38/42 <0.000001	0.07 ± 0.68 (range: −1.56–1.39)	−4.99/40 0.000012

Thirteen depressed participants were taking antidepressants with the following daily dose ranges: citalopram 30–60 mg, mirtazapine 30–45 mg, venlafaxine 75–225 mg. Nearly, all patients with schizophrenia were on atypical antipsychotic medication and the mean chlorpromazine equivalent dose was 401.243 (standard deviation 91.43) mg/day. Three depressed patients were taking antipsychotics: risperidone 2 mg, quetiapine 100 mg, and quetiapine 100 mg. Eight schizophrenia patients were also taking antidepressant medication: citalopram 20–40 mg, fluoxetine 20 mg, mirtazapine 45 mg, and venlafaxine 150–225 mg.

### Negative symptom scales

Ten commonly used negative symptom scales were administered: the negative subscale of the Positive and Negative Syndrome Scale (PANSS) (23); the Snaith–Hamilton Pleasure Scale (SHAPS) (24); Chapman Physical Anhedonia Scale (25); Chapman Social Anhedonia Scale (25); anhedonia subscale of Beck Depression Inventory (BDI) (26); negative symptom subscale of Brief Psychiatric Rating Scale (BPRS) (27); Motivation and Energy Inventory (MEI) social subscale (28); MEI physical subscale (28); Temporal Experience of Pleasure Scale (TEPS) anticipatory subscale (29); and TEPS consummatory subscale (29). Though the PANSS is generally applied to psychotic patients, the PANSS negative subscale has been shown to be a valid instrument in patients with major depressive disorder (30). The BPRS negative symptom scale includes items such as blunted affect, emotional withdrawal, and motor retardation. All scales were self-reported, except the PANSS and BPRS. Higher scores denote more severe negative symptoms, except on the MEI and TEPS. In order to avoid selection bias, we tried to cover all dimensions of negative symptoms: PANSS and BPRS negative symptom subscales are related to impaired cognition, withdrawal, blunted affect, and alogia while the remainder is associated to a greater extent with anhedonia and avolition. It has been suggested that patients with certain mental disorders only suffer a specific type instead of global anhedonia, for instance, anticipatory anhedonia in schizophrenia (29). Many scales have been created for subdivisions of anhedonia, such as the Chapman and TEPS scales used in this study.

### MRI images acquisition and VBM pre-processing

Brain MRI T1-weighted images were acquired by a Siemens Tim Trio operating at 3 T (Wolfson Brain Imaging Centre, University of Cambridge) with the following parameters: slice thickness = 1 mm, repetition time = 2000 ms; echo time = 30 ms; flip angle = 78°; in-plane resolution 2 mm × 2mm; matrix size 240 × 256; bandwidth 2232 Hz/pixel.

Magnetic resonance imaging datasets were pre-processed with VBM8 in Statistical Parametric Mapping (SPM8)^1^ in MATLAB (Math-Works, Natick, MA, USA) following the instructions in the online manual^2^. To begin with, images were segmented into gray and white matter then Diffeomorphic Anatomical Registration through Exponentiated Lie Algebra (DARTEL) was used to increase the accuracy of inter-subject alignment by modeling the shape of each brain using three parameters for each voxel (31). Following DARTEL, templates were generated and registered to the Montreal Neurological Institute (MNI) space by affine transformation. Afterward, all images were non-linearly warped onto the templates and smoothed with 10 mm full width at half maximum Gaussian kernel as suggested in the manual. Possible spatial expansion or contraction during the normalization process was modulated by the Jacobian determinant to preserve the original tissue volumes.

### Principal component analysis and neuroimaging statistics

Principal component analysis simplifies, summarizes, and integrates data along with a reduction in statistical comparisons, thus avoiding inflation of type1 errors. PCA with direct oblimin rotation (32) was performed on all negative symptom scales in all participants via Statistical Package for Social Sciences software (SPSS version 21). Rotation was applied to improve the ease of interpretation, and oblique instead of orthogonal rotation was used due to the presumed association between components. Components with eigenvalues >1 were identified. Participants’ loading scores of these major components were then used for subsequent MRI image analyses in FMRIB Software Library (FSL) version 5.0^3^. A general linear model (GLM) regressed loading scores of the major components onto gray (or white) matter volumes at each intra-cerebral voxel with nuisance covariates of age, gender, and total gray (or white) matter volumes. Four models were regressed for every loading score: gray matter in depressed group; gray matter in schizophrenia group; white matter in depressed group; and white matter in schizophrenia group. In each analysis, a significance level of *P* ≤ 0.05 family-wise error corrected for multiple comparisons was applied using threshold-free cluster enhancement (TFCE) (33). Regions significantly correlated with loading scores were localized by the Harvard–Oxford Cortical Structural Atlas, Johns Hopkins University White-Matter Tractography Atlas, and Cerebellar Atlas in MNI152 space^4^. If two or more regions were significantly related to the component scale in the same patient group and the same tissue type, then inter-regional correlation between the extracted volumes of these areas would also be calculated in the patient and control groups.

### Between-group structural differences related to negative symptoms

After controlling for age, gender, and total gray (or white) matter volumes, regions displaying a significant relationship with principal components in patient groups were used as masks to extract tissue volumes from both the patient and control groups. Using SPSS, between-group differences were assessed via independent, two-sample *T*-tests on these extracted volumes to evaluate the extent of structural group differences related to negative symptoms.

## Results

### Principal component analysis of negative symptom scales

**Figure 1:**
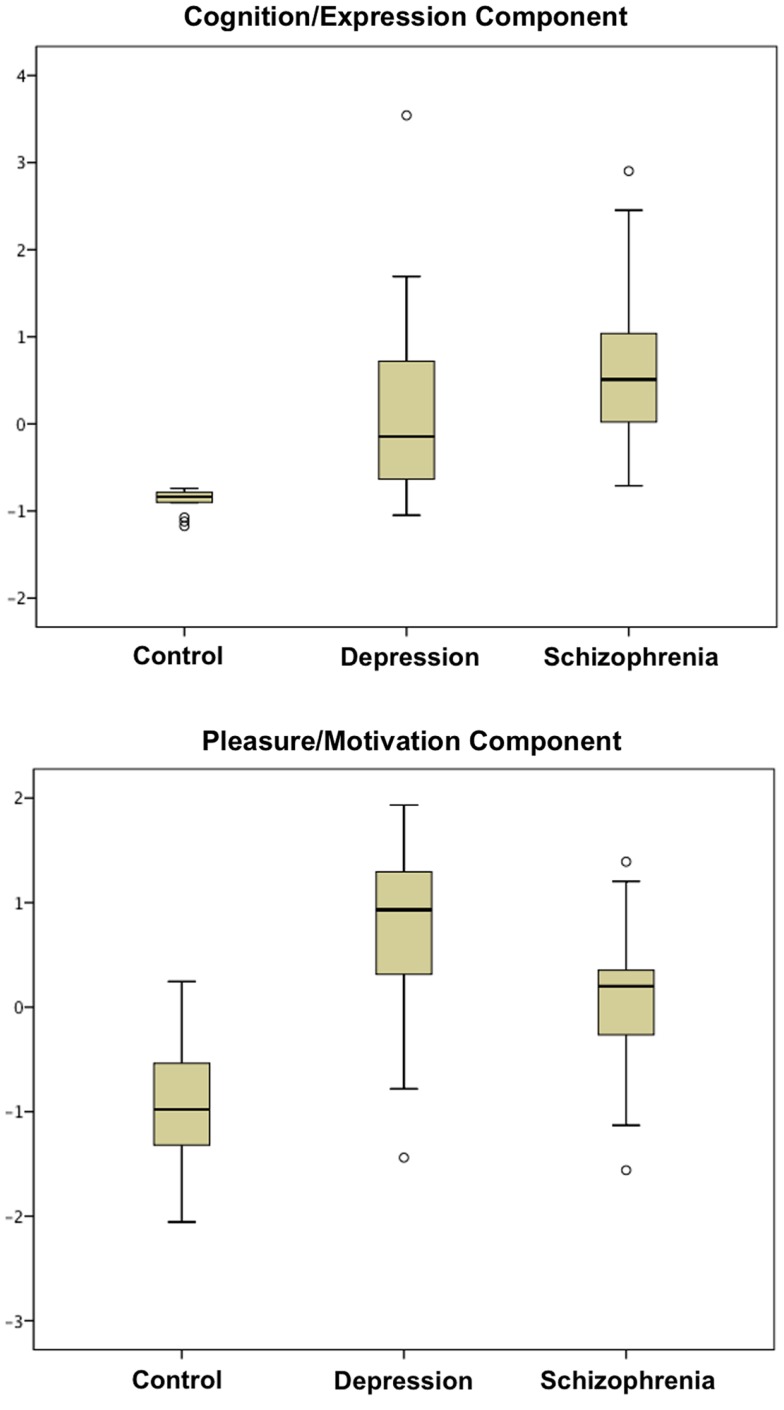
**Boxplots of scores in cognition/expression component and pleasure/motivation component are shown**. Both patient groups are significantly different from the control group in these two components (Table 1).

With a Kaiser–Meyer–Olkin test-statistic value of 0.793 and Bartlett’s test of sphericity with *p * < 0.000001, PCA was adequately completed. Two major components were yielded with eigenvalues >1 (Supplementary Material) collectively explaining 69.93% of the total variance. PANSS negative and BPRS negative subscales loaded more than 0.9 in the pattern matrix onto one component, which was primarily related to impaired cognition, withdrawal, blunted affect, and alogia. The other eight scales loaded onto a second component associated primarily with anhedonia and avolition. We thus identified two principal components, one coding for “cognition/expression” and another coding for “pleasure/motivation” (Tables 1 and 2). Both illness groups expressed each component significantly more than controls (Figure 1). The depressed group scored highest (most impaired) in the “pleasure/motivation” component (*t * = 2.382, *p * = 0.026) whereas the schizophrenia scored highest in the “cognition/expression” component (*t * = 2.627, *p * = 0.016).

**Table 2:** **Pattern matrix of principal component analysis for the 10 negative symptom scales**.

Negative symptom scale	Pleasure/motivation component	Cognition/expression component
Chapman social scale	0.85	−0.22
Chapman physical scale	0.86	−0.06
SHAPS scale	0.73	0.13
MEI social scale	−0.82	−0.04
MEI physical scale	−0.75	0.02
TEPS anticipatory scale	−0.79	−0.08
TEPS consummatory scale	−0.73	−0.18
BDI anhedonia scale	0.70	0.18
PANSS negative subscale	0.04	0.95
BPRS negative subscale	0.05	0.93

### Brain structural correlates of negative symptoms

**Figure 2:**
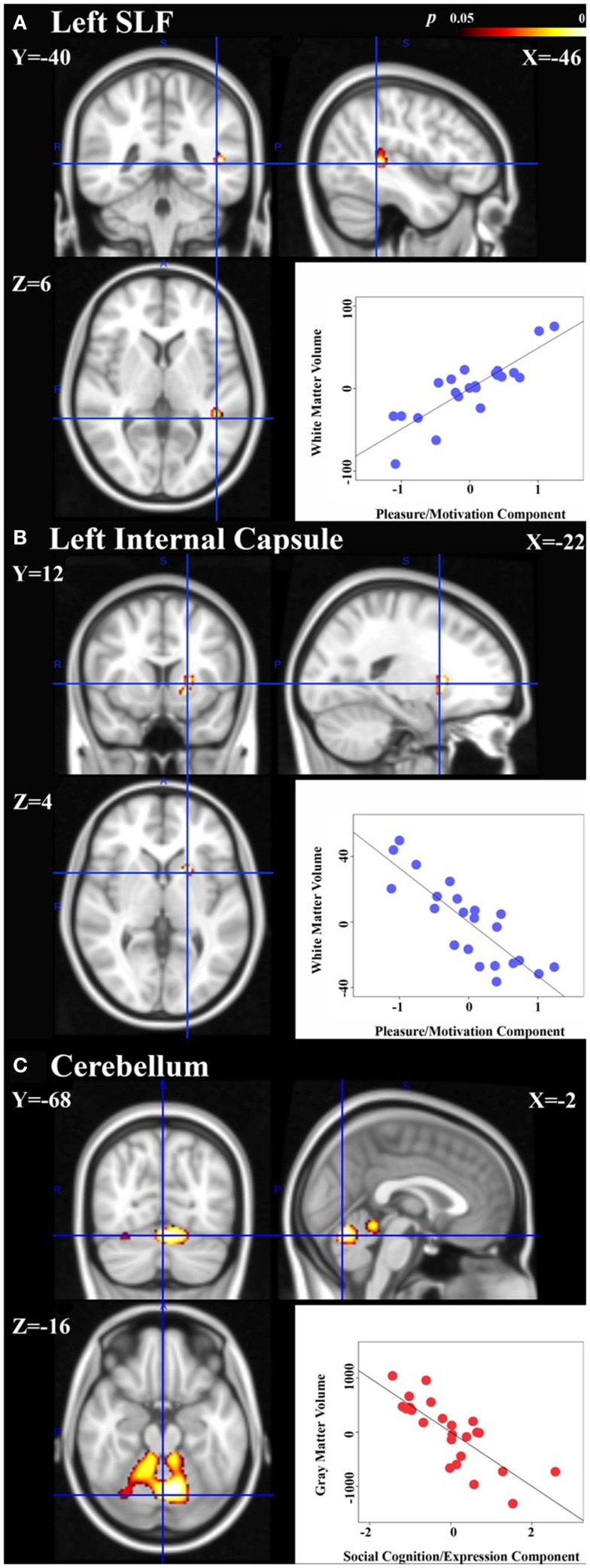
**Neural signatures of negative symptoms in schizophrenia and depression groups are shown**. Significant association of white-matter volumes with the principal component coding for “pleasure/motivation” was found in the schizophrenia group: **(A)** left superior longitudinal fasiculus (SLF) with cluster size = 105 voxels and peak *p * = 0.017. **(B)** Left anterior limb of internal capsule/anterior thalamic radiation with cluster size = 114 voxels and peak *p * = 0.014. **(C)** Significant association of gray matter volumes with the principal component coding for “cognition/expression” was found in the depressed group: cerebellum with a cluster of size = 2212 voxels and peak *p* = 0.002. Peak voxels shown with corresponding Montreal Neurological Institute (MNI) coordinates. Scatter plots: controlling for age, gender, total gray or white-matter volume, partial correlation coefficients (*r*) between the extracted volumes and principal component scores are calculated within group and are shown here [**(A)**: *r* = 0.85, **(B)** *r* = −0.84, **(C)** *r* = −0.79]. Please note that the partial correlation coefficients are expected to be high as the regions in which we quantify that these correlations were defined as being regions with significant associations between volume and principal component expression.

In the schizophrenia patient group, the principal component coding for “pleasure/motivation” was found to be inversely correlated with white-matter volume in the left anterior limb of internal capsule/anterior thalamic radiation and positively correlated with left superior longitudinal fasiculus (Figure 2). Since the principal component coding for “pleasure/motivation” in the schizophrenia group was not significantly correlated with the HAMD-17 score with Pearson correlation coefficient *r * = −0.08 and *p* = 0.741, the assumption of secondary negative symptoms due to depression is not favored. The principal component coding for “cognition/expression” was significantly inversely correlated with bilateral cerebellar gray matter in the depression patient group (Figure 2). These associations were significantly different between groups (Table 3).

**Table 3:** **Group differences in the correlation of the brain regions shown in Figure 2 with the negative symptoms of first two principal components**.

Scale	Region	Group	Fisher *r* to *Z* significance test			
			*r*	v.s. Group	*Z*	*p* Value
Cognition/	Cerebellum	Control	−0.25	Depression	2.51	0.012
Expression component		Depression	−0.79	–	–	–
		Schizophrenia	−0.09	Depression	3.10	0.002
Pleasure/motivation component	Left superior longitudinal fasciculus	Control	0.10	Schizophrenia	−3.45	0.0006
		Depression	−0.05	Schizophrenia	−4.13	0.00002
		Schizophrenia	0.85	–	–	–
	Left anterior limb of internal capsule/anterior thalamic radiation	Control	0.25	Schizophrenia	4.45	0.000009
		Depression	−0.47	Schizophrenia	2.27	0.0232
		Schizophrenia	−0.84	–	–	–

*Controlling for age, gender, and total gray or white-matter volume, partial correlation coefficients (*r*) between the extracted volumes and principal component scores are calculated within group and later transformed to *Z* scores. *Z* scores and *p*-values represent the degree to which the correlations differ between groups*.

**Figure 3:**
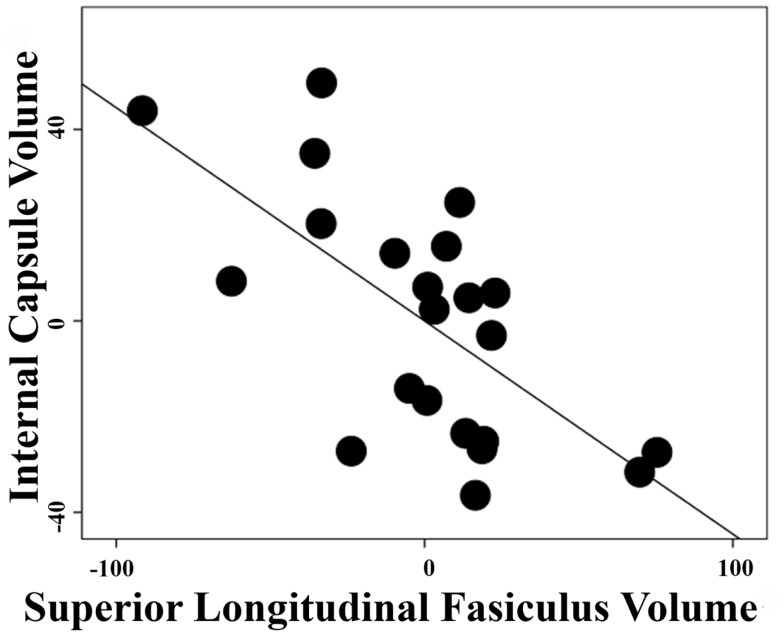
**Inter-regional correlation in schizophrenia group**. Linear regression was performed with a dependent variable of extracted volumes in the left superior longitudinal fasiculus or left anterior limb of internal capsule/anterior thalamic radiation and covariates of age, gender, and total white-matter volumes. Pearson correlation coefficient between the residuals of extracted volumes from these two regions is *r* = −0.66 with *p* < 0.001 in the schizophrenia group.

The correlation between white-matter volume in the left anterior limb of internal capsule/anterior thalamic radiation and left superior longitudinal fasciculus in the schizophrenia group was high with a Pearson correlation coefficient *r * = −0.66 and *p* < 0.001 (Figure 3) but non-significant in the control group with *r * = 0.093 and *p* = 0.696. After Fisher’s *r* to *z* transformation, the group difference of the correlations was significant (*Z* = 2.65, *p * < 0.01). No regions in the gray matter of schizophrenia group or white matter of depression group were significantly correlated with these components.

### Between-group structural differences related to negative symptoms

At a significance level of *P* ≤ 0.05, group differences between controls and patients were revealed in extracted volumes from only one region identified as having a significant relationship to negative symptom scores: the anterior limb of the internal capsule/anterior thalamic radiation (Table 4).

**Table 4:** **Differences between patients and controls in the extracted volumes of areas with significant relationships to the negative symptoms principal components, controlling with age, gender, and total gray or white-matter volume**.

Scale	Area	Compared groups (mean volume)	*t* Value	*p* Value	Degree of freedom
Cognition/expression component	Cerebellum	Control (10179.77)	−0.83	0.409	42
		Depressed (10226.42)
Pleasure/motivation component	Left superior longitudinal fasciculus	Control (539.19)	1.15	0.257	40
		Schizophrenia (508.06)	
	Left anterior limb of internal capsule/anterior thalamic radiation	Control (412.33)	−2.29	0.027	40
		Schizophrenia (420.73)			

## Discussion

### Negative symptoms: Two components, two disorders, and two tissue types

Neuroanalysis following PCA identified neural signatures of two distinct dimensions of negative symptoms in schizophrenia and depression, respectively; that is, a principal component coding for “cognition/expression” in gray matter in depression and a principal component coding for “pleasure/motivation” in white matter in schizophrenia. Therefore, as opposed to a common underlying process proposed by Foussias et al. (34), fundamental discrepancies in the brain structural basis of negative symptoms may exist between different diagnoses and distinct domains. Recruiting nearly 500 participants, a recent study has attempted to develop a new negative symptom assessment for schizophrenia (CAINS: Clinical Assessment Interview for Negative Symptoms). Interestingly, structural analysis of all the items in the CAINS revealed two major elements of negative symptoms similar to our findings, termed by the CAINS researchers as “expression,” a measure of behavioral, social, and emotional expression, and “motivation and pleasure” (3).

Both component scores of depression and schizophrenia patients differ significantly from the controls (Table 1, Figure 1). However, patients showed significant neural correlations with the component, which they did not clinically express predominantly. For instance, the schizophrenia group scored higher in the “cognition/expression” component, with which they failed to demonstrate neural correlates. Further, despite prominent differences in clinical negative symptom severity (Table 1), controls and patients did not differ significantly in all of the extracted volumes within the neural signatures (Table 4). Thus, brain regions in which there are inter-individual differences that relate to symptom severity do not necessarily show differences between groups in average brain volume. This suggests that there may be some neural deficits that contribute to the category of developing either disorder or some separate deficits that influence how severely the illness is expressed.

### The cerebellum in depression

Although initially considered as a motor coordinator, the cerebellum is now known to play a role in emotional regulation and cognition, supported by its widespread connections to the limbic system, the frontal, parietal, prefrontal, occipital, and temporal cortex. The cerebellum is activated in a variety of mental activities, including facial recognition, emotion attribution, theory of mind attributions, directed attention, memory, and empathy (35, 36) and has been referred to as the “emotional pacemaker” (37). Functional mapping of the cerebellum has been established based on functional MRI studies and clinical lesion reports. The posterior lobules VI, VII, and Crus were found to be linked to cognition, the posterior vermis to be associated with emotion and lobules I–V related to sensorimotor function (37). Indeed, the cerebellar cognitive affective syndrome following posterior cerebellar lobe lesion includes negative symptoms such as passivity, blunted affect, and withdrawal (37). Also, early in 1992, Dolan et al. reported increased regional cerebral blood flow of the cerebellar vermis in depressed patients with cognitive disturbance (38). Furthermore, in an fMRI meta-analysis, the cerebellum has been identified as an important area of dysfunction in depression (39). In our depressed group, the bilateral lobules I–IV, V, VI, Crus, and vermis were found to be negatively correlated with the principal component coding for “cognition/expression” corresponding to the functional topography.

### White matter in schizophrenia

Compared to control participants and based on post-mortem and positron emission tomography studies, patients with schizophrenia are suspected to have higher metabolic rates in the superior longitudinal fasciculus and anterior limb of the internal capsule (40, 41) suggesting roles in the pathophysiology of the disorder. The anterior limb of the internal capsule serves as a bridge between the thalamus, cingulate, and prefrontal cortices and thus plays an important role in motivation, reward, and emotion (8). We identified neural correlates of negative symptoms in the superior longitudinal fasciculus III, which connects the supramarginal gyrus with ventral premotor and prefrontal areas (42). The supramarginal gyrus has been reported to participate in execution and mental simulation of action (43). Furthermore, associations between negative symptoms and these two areas have been reported in terms of FA (12) and now white-matter volume in this study. Taken together, this evidence justifies the association of anterior limb of the internal capsule and superior longitudinal fasciculus with a pleasure/motivation domain of negative symptoms. In the schizophrenia group, we demonstrated a significant correlation between extracted volumes in these two regions (*r* = −0.66) (Figure 3), which was non-significant in the controls. Certainly, schizophrenia has been regarded as a disease of disconnectivity (44) and this evidence strengthens that assertion.

### Negative symptom scale development

Encouraged by the National Institute of Mental Health Consensus Development Conference on Negative Symptoms in Rockville, MD, USA, 2005 (45), several attempts have been made in recent years by developing a new negative symptom scale. However, the absence of scales emphasizing the correspondence between scores and structural, or indeed functional, brain changes associated with mental disorders might be accounted for by the diverse findings in the neural correlates of negative symptoms provided here. Despite being difficult to develop, scales reflecting alterations in brain structure and function could be clinically valuable enabling prognosis prediction, disease progress monitoring, and the probing of etiological mechanisms.

Current scales tend to mix several domains of negative symptoms. Nevertheless as shown in our study, it is likely that instead of the whole spectrum, only specific components of negative symptoms correspond to alterations of brain structures. According to our findings, anhedonia and avolition were linked to brain alteration in schizophrenia whereas blunted affect, alogia, withdrawal, and cognitive dysfunction were associated with depression. However, we were unable to distinguish neural correlates of different anhedonia types. Future studies may delve deeper into this topic.

Several limitations should be considered when interpreting our result. We included more participants than some published studies (9, 11, 16, 17), but sample size was still small-to-moderate. Besides, the information regarding disease duration was absent. Furthermore, although we tried to cover all dimensions of negative symptoms by including a large number of frequently used scales, our list of assessments was by no means exhaustive. Other negative symptom scales could be added in future studies. Additionally, the positive correlation in the left superior longitudinal fasciculus with negative symptoms in the schizophrenia group might indicate a compensatory mechanism in response to the deficit. However, we were prevented from providing any cellular explanation of results due to the nature of MRI image analysis. Atypical antipsychotics and cannabis have been associated with brain structural change (46, 47) and were widely used in our patients. Based on two systematic reviews, antipsychotics may alter gray matter volume (48, 49). However, there has been no consensus regarding the directionality of the effect (i.e., increasing or decreasing). Furthermore, we did not find any significant structural difference between patients and controls in the regions in which inter-individual variability of symptoms within patients was associated with inter-individual variability in brain structure. Consequently, we are prevented from determining the exact medication effects. Drug-naïve patients should be recruited in future studies.

In conclusion, in correspondence with the large CAINS study, we identified two major domains of negative symptoms. Neural signatures of these domains were found in depression and schizophrenia. Future research may investigate the association of these regions with negative symptoms. New scales linking negative symptoms with structural brain change are anticipated.

## Author Contributions

Dr. Jie-Yu Chuang performed the analysis and wrote the original draft of this paper. Dr. John Suckling directed the analysis and revised the paper with Dr. Graham K. Murray and Dr. Jie-Yu Chuang. Drs Graham K. Murray, Hisham Ziauddeen, Jenny Spencer, Robert B. Dudas, and Paul C. Fletcher designed the data collection. Dr. Graham K. Murray directed the data collection. Drs Antonio Metastasio, Nuria Segarra, Jenny Spencer, Robert B. Dudas collected the data. Drs Antonio Metastasio, Nuria Segarra, Jenny Spencer, and Tait assisted with preliminary analyses. All authors took part in revising the manuscript and approved the final version.

## Conflict of Interest Statement

The authors declare that the research was conducted in the absence of any commercial or financial relationships that could be construed as a potential conflict of interest.

## Supplementary Material

The Supplementary Material for this article can be found online at http://www.frontiersin.org/Journal/10.3389/fpsyt.2014.00116/abstract

## Footnotes

^1^http://www.fil.ion.ucl.ac.uk/spm

^2^http://www.fil.ion.ucl.ac.uk/~john/misc/VBMclass10.pdf

^3^http://fsl.fmrib.ox.ac.uk/fsl/fslwiki/

^4^http://fsl.fmrib.ox.ac.uk/fsl/fslwiki/Atlases
