# Supplementary material for: Brain Structural Signatures of Negative Symptoms in Depression and Schizophrenia
Source: Front Psychiatry. 2014 Aug 27;5:116. doi: 10.3389/fpsyt.2014.00116 (PMC4145726; doi:10.3389/fpsyt.2014.00116)

Supplementary Material 1. Principal component analysis showing two major components with eigenvalues > 1.

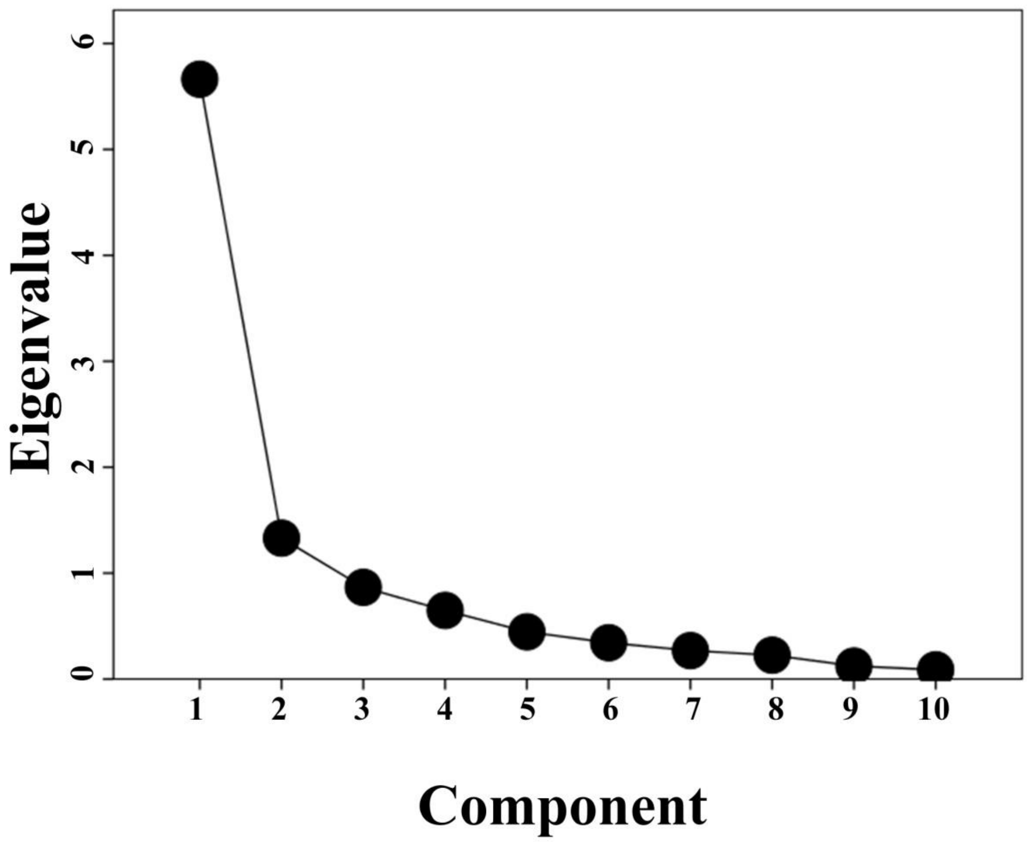

Supplement: Supplementary file 1 [file Image_1.PDF]
